# Understanding orphan and non-orphan adolescents’ sexual risks in the context of poverty: a qualitative study in Nyanza Province, Kenya

**DOI:** 10.1186/1472-698X-13-32

**Published:** 2013-07-25

**Authors:** Milka Juma, Jane Alaii, L Kay Bartholomew, Ian Askew, Bart Van den Born

**Affiliations:** 1 Department of Health Promotion, Maastricht University, Maastricht, The Netherlands; 2 Context Factor Solutions, P.O. Box 27598, Nairobi, Kenya; 3 University of Texas School of Public Health Houston, Houston, Texas, USA; 4 Population Council, P.O Box 17643, 00500 Nairobi, Kenya

**Keywords:** Poverty, Orphan, Non-orphan, Adolescents, Sexual risk behaviour

## Abstract

**Background:**

Some studies show orphanhood to be associated with increased sexual risk-taking while others have not established this relationship, but have found factors other than orphanhood as predictors of sexual risk behaviours and outcomes among adolescents. This study examines community members’ perceptions of how poverty influences adolescent sexual behaviour and outcomes in four districts of Nyanza Province, Kenya.

**Methods:**

Eight study sites within the four districts were randomly selected. Focus group discussions were conducted with a purposive sample of adolescents, parents and caregivers. Key informant interviews were undertaken with a purposive sample of community leaders, child welfare and healthcare workers, and adolescents. The two methods elicited information on factors perceived to predispose adolescent orphans and non-orphans to sexual risks. Data were analysed through line-by-line coding, grouped into families and retrieved as themes and sub-themes.

**Results:**

Participants included 147 adolescents and parents/caregivers in 14 focus groups and 13 key informants. Poverty emerged as a key predisposing factor to sexual risk behaviour among orphans and non-orphans. Poverty was associated with lack of food, poor housing, school dropout, and engaging in income generating activities, all of which increase their vulnerability to transactional sex, early marriage, sexual experimentation, and the eventual consequences of increased risk of unintended pregnancies and STI/HIV.

**Conclusion:**

Poverty was perceived to contribute to increasing sexual risks among orphan and non-orphan adolescents through survival strategies adopted to be able to meet their basic needs. Policies for prevention and intervention that target adolescents in a generalized poverty and HIV epidemic should integrate economic empowerment for caregivers and life skills for adolescents to reduce vulnerabilities of orphan and non-orphan adolescents to sexual risk behaviour.

## Background

Studies in Sub-Saharan Africa show adolescents of age 15–19 are vulnerable to socioeconomic deprivations and sexual and reproductive health risks that are often associated with poverty [1]. These include teenage pregnancy, school dropout, early marriage, and HIV infection. The HIV epidemic is gendered with girls constituting three quarters of HIV infections in this age group [1].

In Kenya, the HIV prevalence rate among adults (15–49) is 7.4%, with marked regional variations ranging from 0.8% in North-Eastern Province to 14.9% in Nyanza Province [2]. Nyanza Province is located along the shores of Lake Victoria and is the focus area of study for this paper. The region is predominantly inhabited by the *Luo* ethnic group with the highest HIV prevalence of 20%, three times the national average [2,3]. The province bears one-third of the national HIV/AIDS burden in Kenya [2].

Among Kenyan adolescents aged 15–19, females are 3.5 times more likely to be infected with HIV than males. Nyanza Province has the highest HIV prevalence rate at 8% with young women nearly four times as likely as their male counterparts to be infected (11% vs. 3%). Other provinces have rates between 1% and 3%. The province also has the highest teenage pregnancy rate of 27% in Kenya [3]. Factors associated with HIV and pregnancy risks for adolescents include poverty, orphanhood [4] and low levels of education [5].

About half of the Kenyan population (47%) lives below the poverty line, on less than one US dollar per day [6,7]. Nyanza Province has the highest population in Kenya living below the poverty line at 65%, with marked constituency (political boundaries represented by a Member of Parliament) differences ranging from 43% - 80%. The province contributes 19% of the total national poverty [6,7]. The main economic activities in the region include: fishing, subsistence farming, and small-scale trade in various fields. Nyanza also hosts many landing beaches in various districts including Suba, Kisumu, Homa Bay, and Migori; the higher the number of landing beaches, the higher the HIV prevalence rate. Sentinel surveillance statistics show that Suba, which has the most landing beaches, has the highest prevalence rate of 26.3% followed by Kisumu at 18.5% [8,9].

Nyanza Province hosts the largest proportion of orphans in Kenya at 19% [2]. The generalized HIV epidemic and high levels of poverty in Nyanza Province may enhance orphan vulnerability to sexual risk behaviours with negative outcomes [10-12]. Studies conducted in southern Africa have shown orphaned adolescents at heightened risk of HIV infection [13-17], STI infection [13,14], unintended pregnancy [13,15-19] and transactional sex [16,20,21], especially those living in poverty. These studies were mainly conducted in South Africa and Zimbabwe, countries with much higher HIV prevalence rates and socio-cultural contexts different from that of Kenya. Contrary to the southern African findings, a study conducted in Nyanza province of Kenya did not find any association between sexual risk behaviour and orphanhood [22]. The conflicting result from Kenya compared to South Africa and Zimbabwe prompts further investigation to consolidate the evidence on pathways to sexual risk behaviour among orphan and non-orphan adolescents.

This paper explores community members’ perceptions of factors that influence behaviours of orphan and non-orphan adolescents that may predispose them to STI/HIV infections and teenage pregnancy. A better understanding of such factors will inform policy and interventions to improve adolescent general well-being and sexual and reproductive health specifically.

## Methods

### Study design

This study used focus group discussions (FGDs) and key informant interviews (KIIs) to gather perceptions from study groups/participants. Focus group discussions included parents, caregivers and adolescents. In this study an adolescent was defined as a child of age 10–17 and an orphan as a child aged10-17 who has lost one or both parents. A parent was defined as a community member with a child aged 10–17 and a caregiver as a community member living with and caring for an adolescent orphan or non-orphan aged 10–17 who was not their child; however, they may also have their own children in the same age group. In conducting and reporting our study we combined parents and caregivers into one category called ‘caregivers’. A key informant was defined as a community member or non-member who lived in or worked closely with the community and knew it well enough to discuss issues affecting the people, orphan households, orphans and adolescents in general.

The two methods were used to give broad community perspectives on influences of poverty on adolescent sexual behaviour and for data triangulation to establish validity of findings. Including both caregivers and adolescents provided two different perspectives to add to validity of findings.

### Setting and sampling

The study was undertaken in four districts of Nyanza Province: Kisumu, Homa Bay, Migori and Suba. The districts were chosen to provide additional information that could further inform a Cash Transfer (CT) project [23,24]) that was to be piloted in the districts to address the socioeconomic needs of orphaned and vulnerable children (OVC) households. The CT is a government social protection program for OVC that provides regular and predictable cash transfers to extremely poor families living with OVC to encourage fostering and retention of OVC within their families and communities, and to promote their human capital development. Using stratified sampling of communities, two constituencies were randomly selected from each of the four districts. For each constituency, a location (an administrative unit) was randomly chosen, within which one sub-location (the smallest administrative unit) was then chosen randomly, giving a total of eight sub-locations for the study sites. The participants in focus groups were chosen purposively to make sure that both adolescent boys and girls were represented equally and that also male caregivers were represented. With awareness of and sensitivity to the generalized HIV epidemic and poverty, we invited adolescent and caregiver participants to participate with attention to age and sex only. We did not choose based on HIV status. Pre-test experience indicated that female caregivers were more available than their male counterparts.

A total of 14 FGDs were conducted with147 participants, eight with 78 adolescents ages 14–17 (four with boys and four with girls) and six with 69 caregivers in two age groups of 25–49 and 50 years and above, distributed by gender. Of the six caregiver FGDs, four were for females and only two for males. Each FGD had 10–12 participants. Thirteen KIIs were also conducted with two school-going male and female adolescents aged 15 and 16 years respectively, five community leaders, one District Children’s Officer (DCO), two Area Advisory Council representatives, and three health care providers.

### Procedures

The FGD and KII guides explored the perceptions of the community on factors that affect sexual behaviours of adolescent orphans and non-orphans, and caregiver communication with adolescents on teenage pregnancy, sexually transmitted infections (STIs) including HIV and prevention. We translated the guides into Kiswahili and the local language, *Dholuo* and the first author pre-tested them with similar respondents in communities neighbouring the study sites to assure question clarity.

Community leaders worked with the study team to recruit participants through a purposive approach to ensure that we included the types of participants described above. The FGDs and KIIs were conducted by ten trained and experienced qualitative interviewers who also obtained oral informed consent and assent from all adult and adolescent participants respectively. Caregiver consent was sought first for the selected adolescents to participate before seeking the adolescent’s assent. Discussions and interviews were conducted in Kiswahili, Luo or English depending on participants’ language preference. All discussions were audio-taped with participants’ consent and lasted 1 hour to 1 hour and 45 minutes. The FGDs in each study site ran concurrently at a central location within the community. Activities were conducted between 10 a.m. and 3 p.m. The KIIs were conducted at a convenient place for the respondents with audio privacy.

The study was approved by the institutional and ethical review boards of the Population Council and the Ministry of Science and Technology.

### Data analysis

The qualitative approach to data analysis was deductive from the standpoint that we were looking for specific relations between orphanhood and poverty rather than a more constructivist narrative [25]. The audio-taped FGDs and KIIs were transcribed verbatim and translated into English for those conducted in Kiswahili and the local language. The first author read all FGD transcripts, and developed an initial coding scheme that was reviewed and refined by the co-authors. ATLAS.ti 5.2 software was used for line-by-line coding [26]. These codes were further grouped into families and retrieved as emerging themes and sub themes. The following themes relating to poverty and risky sexual behaviour emerged from the FGDs and interviews and are reported below: poverty facilitative factors, and sexual risk facilitative factors; lack of basic needs, poor housing, school dropout, engaging in income generating activities, transactional sex and early marriage.

## Results

### Background characteristics

Of the 147 participants 53% were adolescents and 47% caregivers. Table 1 presents the characteristics of participants. There were more females than males among caregivers with 48% aged 30–49 and the rest aged 50–72. Additionally, the majority was married or widowed with primary or secondary education. All adolescents were aged 14–17 with 53% males, all unmarried and currently schooling with the majority in primary and more than one-third in secondary.

**Table 1:** Demographic characteristics of participants

**Characteristics**	**Study categories by data collection methods**		
	**FGD**		**KII**
	**Parents/caregivers**	**Adolescents**	
	**(n = 69)**	**(n = 78)**	**(n = 13)**
**Sex**			
Male	24 (35%)	37 (47%)	9 (69%)
Female	45 (65%)	41 (53%)	4 (31%)
**Age group**			
14–17	-	78 (100%)	2 (15%)
30–49	33 (48%)	-	7 (54%)
50–72	36 (52%)	-	4 (51%)
**Highest level of education**			
No education	11 (16%)	-	-
Primary	27 (39%)	48 (62%)	2 (15%)
Secondary	25 (36%)	30 (38%)	5 (39%)
Post Secondary	6 (9%)	-	6 (46%)
**Marital Status**			
Single	-	78 (100%)	2 (15%)
Married	42 (61%)	-	11 (85%)
Widowed	27 (39%)	-	-
**Categories of key**			
**Informants**			
Adolescents	-	-	2 (15%)
Community leaders	-	-	5 (39%)
Child welfare workers	-	-	3 (23%)
Health care providers (HCP)	-	-	3 (23%)

### Factors that facilitate poverty

Participants in all focus groups and interviews described poverty as inability to provide for basic needs, and emphasized it as a key facilitative factor of adolescent sexual risk behaviour. Adolescents, caregivers, community leaders, and service providers discussed this concept extensively. Caregivers described five factors that exacerbated poverty in their communities. First, many AIDS-related deaths among adults that burden their extended families with the care of orphans for whom they could not adequately provide for:

“Parents have really died mainly due to AIDS related illnesses and left many orphans behind for us to care for. We also have our children who are already burdensome. We are unable to care for them well. Education is very expensive; to educate your children and leave the orphans is a challenge. They both need equal opportunities. We parents and caregivers have been unable to care for them well.” (Male caregiver, FGD)

Additional factors include unemployment, low income, high cost of living, low farm produce due to unreliable rainfall and poor farming techniques. Also reported were lack of markets, low prices of farm produce when harvest is good, and poor roads.

“There is starvation in our village. If you are not working and only depend on farming, then there is no way you can survive because farms are not doing well.” (Female caregiver, FGD)

“You can be a farmer but still be poor because we sell what we produce at throwaway prices.” (Male caregiver, FGD)

“Some parents/guardians have farms but they lack farm implements so some of the farms are not cultivated. Such parents should be helped by the government to expand farm production for food security in orphan households.” (Female adolescent, FGD)

### Factors that facilitate adolescent sexual risks

#### Lack of basic needs

Participants across all categories reported poverty as a hindrance to caregivers’ provision of children’s basic needs, which prompted some adolescents to adopt varied survival strategies. The most commonly and extensively discussed basic needs were lack of food, and school requirements such as school fees, uniform, shoes, and stationery. Other basic needs included clothes, underpants, toiletry supplies and poor housing in low income urban areas:

“Many adolescents, both orphans and non-orphans, who live in poor households lack many basic necessities. Many orphan households are poor and this will make them leave their household to seek self support elsewhere.” (Male adolescent, KII)

“Poverty in a family can make an orphaned girls resort to transactional sex to supplement the little food the parents/guardians can afford.” (Female adolescent, FGD)

“Some boys move from one household to another to herd cattle and in order to get food.” (Male adolescent, FGD)

#### Poor housing

Poor housing in low income urban areas was reported as a sexual risk factor for adolescents especially by female caregivers. They reported that poor housing, had limited audio and visual privacy; exposing children to sexual intimacy by their parents, caregivers or neighbours that motivated sexual experimentation:

“In this place (urban slum) most of us live in one-roomed rental houses mainly joined structures (*landi*) without ceiling, partition or with poor partition material. You have no choice but to share the single room with the children who sleep on the floor. The children will ‘watch you when you are with their father’, so we usually ask them to play outside until we call them to prepare their sleeping space. In the course of waiting outside, some children end up engaging in unprotected sex, drugs or alcohol.” (Female caregiver)

Housing in urban informal settlements thus posed a challenge to adolescent sexual risk prevention.

#### School dropout

School dropout as an outcome of poverty, as a survival strategy, and as a sexual risk factor was reported in nearly all adolescent FGDs, a half of caregiver FGDs and at least a half of KIIs. Adolescents dropped out of school due to lack of school requirements, food, and the need to work to support family basic needs:

“Some adolescents, both orphans and non-orphans from poor families drop out of school due to lack of money for school requirements such as school fees, uniform including shoes, inability to concentrate due to hunger, frequent lateness and absenteeism that result in loss of interest and eventual dropout.” (Female Adolescent, FGD)

Adolescents also perceived orphans to be more susceptible to school dropout than non-orphans:

*“*Orphans are more at risk of dropping out of school, for example, some paternal orphans are forced by circumstance to drop out of school to help the mother with casual work for the family to get money for household food.” (Male adolescent, FGD)

Caregivers reported many parents’ inability to cater for their children’s secondary school education, resulting in school dropout thus predisposing them to risks faced by out of school adolescents.

“Many parents in this region cannot afford to take children to secondary school. Such children become idle and begin to influence each other into sex.” (Male caregiver, FGD)

#### Engaging in income generating activities

Adolescents, caregivers and community informants reported that, to meet basic needs, some adolescents engage in varied income generating activities (IGAs). The most commonly discussed IGAs included fishing, bicycle taxi services, herding and casual farm work for boys while girls engaged in domestic live-in work (household chores for pay) away from home.

Adolescents reported that boys who engaged in IGAs used their earnings to entice girls into sex, go to discos and video shows, buy alcohol and drugs, and engage in other behaviours that were thought to increase sexual risk outcomes such as teenage pregnancies, STIs or both.

They described domestic work as increasing girls’ vulnerability to sexual abuse and exploitation by the male members of such households:

“Some parents can remove a child from school to work as a domestic worker to bring money home so that the parent can buy food for the family. This puts the girl at risk of pregnancy or contracting STI/HIV as she may be sexually abused or exploited by some male members of the household she works for.” (Male caregiver, FGD)

#### Transactional sex

All participants extensively discussed transactional sex as an important sexual risk behaviour associated with poverty. Orphans and non-orphans from poor families were both at risk of engaging in transactional sex to get money to meet their basic needs:

*“*Sometimes orphans who live in a poor household and especially the girls who have the responsibility to provide for their daily meals ends up in engaging in sex to get money for food and other basic needs.” (Male Adolescent FGD)

“Adolescents who have parents but live in poverty engage in transactional sex because of the problems they face. They will copy their friends whom they see engaging in sex for money to make ends meet.” (Female adolescent, FGD)

Orphaned and sometimes non-orphaned males were reported to engage in transactional sex with older women and widows to meet basic needs such as food and school fees:

*“*A male orphan who lacks school fees or pocket money may decide to stay with a widow who has money so that she pays for his school fees.” (Male Adolescent, FGD)

Transactional sex is also facilitated by the IGA environment in which adolescents operate, such as fish landing beaches, as expressed by male adolescent FGD participant:

“Beaches serve as money minting points where fishermen, fishmongers and middlemen have a lot of money. For boys involved in the fish trade, older female fishmongers, some of whom are widows, entice them with sex so that they can sell to them fish cheaply. This has led to high rates of HIV infection in the beach communities”.

#### Early marriage

Poverty was reported to motivate orphan and non-orphan girls into early marriage that was either voluntary or forced by caregivers:

“If a family is living in abject poverty and they have daughters, some parents have been reported to marry them off even before age 18 to get dowry to sustain the family economically. Such cases are reported to my office, and do affect both orphans and non-orphans and puts them at risk for early pregnancy or contracting STI/HIV.” (DCO, KII)

Poverty compelled orphan and non-orphan girls to early marriage to escape the challenges they faced such as caregiver inability to cater for their school requirements including other basic needs, with expectations that their spouses would cater for their basic needs. Caregivers and adolescents reported an additional challenge of teenage pregnancy and fear of consequences.

“An orphan or non-orphan girl who is suffering can decide to go and get married to a better off man so that her misery comes to an end” (Male adolescent, FGD)

## Discussion

In this qualitative study poverty was found to provide the context for transactional sex, early marriage and sexual experimentation among orphans and non-orphans. Lack of basic needs was perceived to lead directly to engaging in these behaviours as a survival strategy to meet such needs or indirectly through school dropout to engage in IGAs whose environments and proceeds predisposed adolescents to transactional sex. School dropout was the only factor perceived to affect orphans more than non-orphans.

Various studies have suggested that AIDS-related illness or death of people in the productive age group increases household poverty, food insecurity and unemployment [27-29]. In our study, these factors were identified to predispose adolescents to risky sexual behaviour. Thus, pregnancy and STI/HIV prevention and care interventions ought to integrate strategies that increase food security and empower caregivers economically to be able to meet adolescent basic needs. This helps to explain why food was the top basic need that contributed to sexual risk behaviour among adolescents irrespective of orphan status.

Our study identified poverty as a hindrance to adolescent orphans’ and non-orphans’ access to basic needs, results that are consistent with other studies [28,30]. The findings show how poverty contravenes various basic child rights of adolescents leading to sexual risk behaviours.

Early marriage is one of the traditional practices prejudicial to the health of children that elevates adolescent girls’ risk of HIV infection because of the HIV status of their husbands and frequency of intercourse [31-34]. Early marriage among girls is common in many Sub-Saharan African countries including Kenya, which predisposes them to varied reproductive health risks such as early or teenage pregnancy, cervical cancer and sexually transmitted infections including HIV, spousal sexual and physical violence, and less likelihood to use prevention of mother to child transmission or antiretroviral therapy services [31].

Our findings also suggest that poverty hinders adolescent access to education due to lack of food and school requirements leading to school dropout. Non-schooling has been shown to limit adolescent access to sexual and reproductive health information that contributes to their susceptibility to sexual risks [35].

The results show that by engaging in IGA, adolescents expose themselves to environments that predispose them to sexual risks. Additionally, domestic work exposes girls to sexual abuse and exploitation as well as transactional sex. For boys engaged in fishing, the context in which small-scale fishing industry is conducted predisposes them to risky sexual behaviour. Examples include the fish-for-sex culture and the mobility of the fishers that make the fishing communities highly susceptible to STI/HIV infection [8,9,36,37].

Risky sexual behaviour such as transactional sex expose adolescents to unplanned pregnancy and STI/HIV infections that hinder the goal of preventing and mitigation HIV/AIDS [15,19,35,38,39] . Transactional sex often occurs with older partners [20,40] and the power imbalance in such exchange reduces adolescent ability to negotiate safe sex, thus predisposing them to unprotected sex [41-44].

The lack of a clear difference between orphans and non-orphans in the perceived influence of poverty on sexual behaviour and health outcomes suggests that poverty in itself may be a more influential factor than orphanhood. Our findings on poverty as a predisposing factor for adolescents’ sexual risks is corroborated by another study in the same province that found low household socioeconomic status and educational attainment to be positively associated with transition to sexual activity, marriage and pregnancy among adolescent girls [45].

The results indicate a need to review the existing poverty reduction strategies and interventions, and the extent to which they address adolescents’ needs. Operations research would help to identify ways to address poverty to reduce sexual risks among adolescent orphans and non-orphans. Our study findings also reveal that while orphanhood may exacerbate vulnerability to risky sexual behaviour, an analysis of the poverty context may provide a better understanding of why both orphan and non-orphan adolescents may be at risk for STI/HIV infection and teenage pregnancy.

Our study has various limitations that should be considered when interpreting the results. It is possible that participants’ selection may have been biased as community leaders assisted in the recruitment of participants. Additionally, it is possible that poverty may not have been discussed exhaustively as only one wave of data collection was conducted. We did not use an iterative approach to data collection. Such an approach would have allowed us to learn from each stage to focus questions at the next stage; instead, we conducted the FGD’s concurrently. Despite these limitations, our findings generate useful information that should refocus attention to the generalized poverty in the province and inform policy and programming on sexual risks for adolescents.

## Conclusions

Our findings highlight pathways by which poverty contributes to sexual risk behaviour among both orphaned and non-orphaned adolescents through their lack of basic needs, school dropout, engaging in IGA that make adolescents susceptible to sexual experimentation, transactional sex, and early marriage that expose adolescents to unplanned pregnancies and STI/HIV risks. Prevention policies and interventions targeting adolescents in communities with generalized poverty and HIV should encompass: i) economic empowerment for all parents and caregivers, aimed at reducing household economic vulnerabilities; ii) life skills targeting adolescents, irrespective of orphan status; and iii) strengthening the existing social protection system implemented by the government that provides regular and predictable cash transfers to extremely poor households living with OVC to encourage their fostering and retention within their families and communities, and to promote their human capital development. These government protection services include school enrolment and attendance, household nutrition and food security, improved reproductive health services for adolescents, and increased civil registration of children and caregivers, thus addressing most of the basic needs that predispose adolescents to sexual risk behaviour.

## Abbreviations

CT: Cash Transfer; DCO: District Children’s Officer; FGD: Focus Group Discussion; IGA: Income Generating Activities; KII: Key Informant Interview; OVC: Orphan and Vulnerable Children.

## Competing interest

The authors declare that they have no competing interests.

## Authors’ contributions

MJ made substantial contributions to the conception and design, drafted data collection instruments, trained data collectors, participated in data collection, analysis and interpretation, drafted this paper, and was responsible for submission. JA contributed to the drafting of the paper and revision of the version to be submitted. LKB made substantial contributions to data analysis and interpretation, and revised the first draft of the paper and version to be submitted. IA made substantial contributions to the conception and design, and edited the draft versions of the data collection instruments, and revised the draft paper and version to be submitted. BVB made substantial contributions to the data analysis, interpretation, edited draft versions of this manuscript and the version to be submitted. All authors read and approved the final manuscript.

## Pre-publication history

The pre-publication history for this paper can be accessed here:

http://www.biomedcentral.com/1472-698X/13/32/prepub
